# The Rhythms of Scholarly Publication: Suggestions to Enhance Bibliometric Comparisons Across Disciplines

**DOI:** 10.3389/frma.2022.812312

**Published:** 2022-01-25

**Authors:** Anthony J. Olejniczak, William E. Savage, Richard Wheeler

**Affiliations:** Academic Analytics Research Center (AARC), Columbus, OH, United States

**Keywords:** bibliometrics, higher education research, academic publishing, academic disciplines, research evaluation

## Introduction

The need for research universities to understand expectations for scholarly publishing across a wide array of disciplines is vital to their self-understanding and to decision-making arising from that understanding. Administrators, staff, and faculty members serve on college-level executive committees, provost-level promotion and tenure committees, and committees that oversee campus-wide recognition of research excellence. They engage in processes that allocate research funding in response to competing proposals from several fields, or that authorize and oversee the development of cross-disciplinary programs. They develop strategic plans shaped by an awareness of the strengths, needs, opportunities, and vulnerabilities of units across the university. In these processes, they are called upon to assess the scholarly output of faculty in disciplines representing a wide variety of publication cultures. They see the vitas of faculty in many fields and career stages, and come to realize, for example, how different the CV of a chemist is from the CV of a historian. Over time, experienced administrators and faculty develop an intuitive understanding of these differences, but even that understanding can be strengthened and extended by data that reflect essential dimensions of discipline-based publication patterns.

Different disciplinary cultures have different understandings about the use of bibliometric data to influence decision-making, some embracing it, others resenting it. But all indicators make it clear that the role of bibliometrics in shaping the future of universities—from the department level to central administration—is increasing. Research universities are in a time of considerable flux, with pressures coming from factors as various as enrollment patterns; an intense emphasis on diversity; changing public perceptions of the nature and value of education; political interventions; shifts in funding patterns; research costs not covered by external funding; high tuition and student debt; and an ongoing debate within universities about the fundamental purposes of research and education. It is more than ever important to be certain that administrative decisions in this highly charged conceptual and funding environment reflect accurate internal assessments of research productivity across the disciplines that constitute each university. Here, we suggest ways to refine that understanding based on recently produced data tables of publication patterns in 170 disciplines (https://osf.io/myaut/); these tables, and their methodology, have been reproduced as Supplementary Materials to this opinion.

## There's More than Journal Articles

### Bibliometric Evaluation Should Be Conducted With an Appreciation of Disciplinary Publishing Modalities: Disciplinary Literatures Are More Than Just Journal Articles

Peer-reviewed journal articles are common in nearly every research-focused academic unit, but conference proceedings, books, chapters in edited volumes, and other publication types are also important in many disciplines, sometimes as the primary means of dissemination. Nonetheless, comparative displays of research outputs often focus solely on journal articles (and citations to journal articles). This is particularly true among widely consulted (if often maligned) university ranking schemes, effectively penalizing universities whose faculty disproportionately practice in disciplines that favor other modes of knowledge dissemination. QS World University Rankings, for example, includes only citations to journal articles in its research impact component, noting in their methodology that normalizing citation counts “ensures that universities that are strong in the humanities or social sciences have almost as good an opportunity to feature in our results as those strong in the sciences” (QS Top Universities, 2021). US News and World Report rankings include book publications, but journal articles and citations are given twenty times greater weight (Morse and Vega-Rodriguez, 2020). Many bibliometric studies focus exclusively on journal articles as they relate to other indicators, such as research funding (e.g., Garrison et al., 1992) or patent production (e.g., Teitel, 1994). Studies focused on the relationship between journal articles and econometric patterns are informative in many disciplines (largely STEM areas), but reliance on these studies to guide bibliometric evaluations across a broader range of disciplines underscores the central premise of this Opinion: the publications of scholars in disciplines likely to win fewer grants, generate fewer patents, and produce fewer journal articles are not adequately represented in many bibliometric analyses and data sources. Some bibliometric databases represent curated lists of journal titles (e.g., Web of Science, Scopus), but often are biased in favor of archiving the published works of STEM scholars (e.g., Orduna-Malea et al., 2019).

Discipline-normalization techniques and disciplinary differences in citations and journal article publication counts are widely discussed in the scientometric literature (reviewed recently by Waltman and van Eck, 2019), ranging in complexity from simply categorizing journals into areas of inquiry to sophisticated article-level classification algorithms. Other aspects of journal article publications have also been studied across disciplines (e.g., authorship order: Fernandes and Cortez, 2020). Common to many discipline-normalization techniques is that each journal or article is classified into one or more disciplines (an illustrative comparison of two journal-level classification techniques is given by Perianes-Rodriguez and Ruiz-Castillo, 2017), and in some normalization systems the researcher is the unit classified (e.g., Abramo and D'Angelo, 2014). Applying a journal-, article-, or person-based classification approach to academic department evaluation in a university administrative context, however, is potentially problematic. A faculty member whose research program focuses on developing new quantitative analytical methods, for example, may publish mainly in journals classified as “statistics,” while this person's academic appointment is in a psychology department. Classifying the scholar's discipline as “statistics” based on their bibliometric profile fails to capture the author's role as a member of the psychology department—a role that is critical to the proper contextualization of the department's research as the product of an academic unit. The data underlying this analysis (available as Supplementary Materials) are classified by the academic unit of the author, which we believe is the ideal discipline-normalization technique for the case of academic department evaluation.

**Figure 1:**
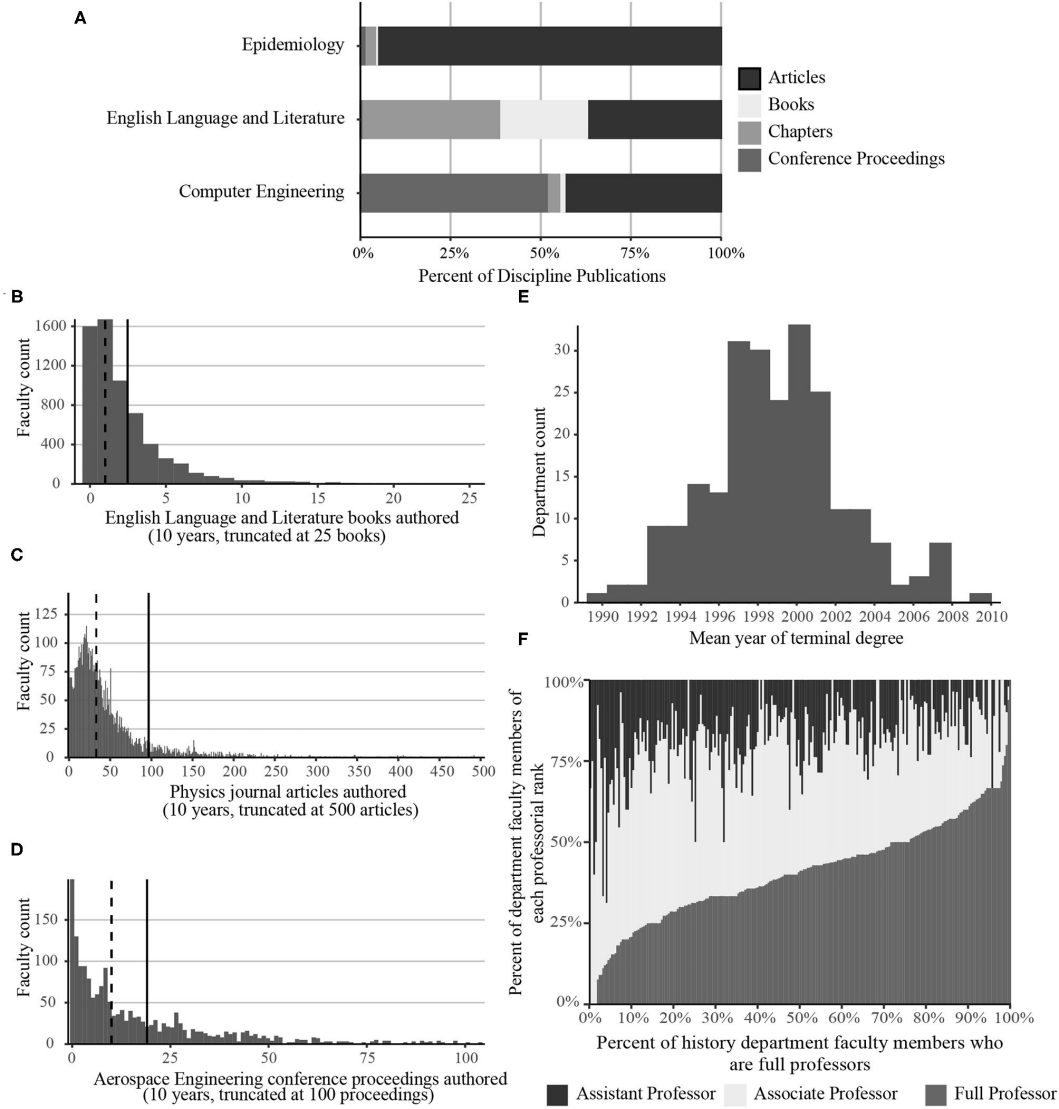
**(A)** Percent of the total published literature represented by four research dissemination modalities (journal articles, books, chapters in edited volumes, and conference proceedings) in three academic disciplines with divergent publishing practices. **(B–D)** Publication count distribution (2010–2019) by number of faculty members for: **(B)** books authored by faculty members in departments of English Language and Literature; **(C)** journal articles authored by faculty members in departments of Physics, and **(D)** conference proceedings authored by faculty members in departments of Aerospace Engineering. In **(B–D)**, solid vertical lines represent the discipline mean and dashed vertical lines represent the discipline median. **(E,F)** Distributions of department mean year of terminal degree and department professorial ranks in History departments at US Ph.D. granting universities. In **(E)**, the mean year of terminal degree for each History department was calculated, and the departments were plotted as a histogram. In **(F)**, each History department is a vertical line showing the percent of faculty members in that department who are assistant, associate, and full professors; departments are sorted from left to right on increasing percent of full professors in the department.

Three extreme examples of disciplinary publishing patterns, culled from our sample of 170 disciplines, appear in Figure 1A: epidemiologists publish mainly journal articles, more than 60% of English language and literature publications are books and book chapters in edited volumes, and conference proceedings are published more often than any other mode of dissemination among computer engineers. *Bibliometric evaluation using only journal articles fails to capture more than 50% of the published works in 26 of 170 disciplines over a 10-year timeframe, almost all of which are in the humanities* (Supplementary Table 1). Many bibliometric databases employ discipline-normalization to account for the relatively fewer journal articles authored by humanists and some social scientists, but journal article count normalization still leaves a large portion of non-article publications totally unaccounted for. In addition to book-oriented disciplines, publishing patterns in STEM disciplines are also incompletely understood if journal articles are disproportionately weighted or represent the only publishing modality. Computer Scientists and Electrical Engineers, for example, publish more conference proceedings than articles (Supplementary Table 1). Likewise, book chapters constitute more than 25% of the literature in many Humanities, Social Sciences, and Education disciplines.

## What is a Reasonable Publishing Expectation in this Discipline?

### Evaluating Publishing Output Based on the Discipline's Mean Contributes to Unrealistic Publishing Expectations, Unduly Influenced by a Minority of Prolific Scholars

A sometimes-overlooked characteristic of bibliometric data is that skewed distributions are common, as Lotka (1926) demonstrated in an early bibliometric study. Publication counts evince a long tail due to a small number of researchers publishing an exceptional volume of work. Three such distributions appear in Figures 1B–D, based on data from Supplementary Table 2. The mean number of publications is more than twice the median value in some disciplines (e.g., Figure 1C). Schmoch (2020) proposed an adjusted mean value for ranking research units on journal article citations. Although Schmoch did not explicitly extend this citation-focused method to publication counts, Supplementary Table 2 demonstrates that mean publication counts are also influenced by prolific outliers. We propose that a discipline's median publication count is a more appropriate measure of publication activity than the mean count for bibliometric evaluation. The median is easily calculated, widely understood, and better addresses questions posed by evaluators of bibliometric outputs across disciplines (e.g., “How many publications does a scholar in this discipline typically publish?”).

## The Rhythm of Publication

### Bibliometric Data in an Evaluative Context Should Normalize for the Publication Rhythms Specific to Each Discipline by Choosing a Timeframe That Captures the Most Recent Published Work of Most Scholars in That Discipline

The pace, or rhythm, of publication varies between disciplines—sometimes substantially—even within the same broad area of research. Discipline-specific rhythms reveal an important and, we believe, often overlooked aspect of bibliometric comparisons: the timeframe examined has a profound effect on bibliometric counts. Supplementary Table 3, for instance, shows 81% of historians have authored at least one book in the most recent 10-year period. Over a 5-year period, the percent of historians with a book publication drops to 56%, and over 3 years only 42% have authored a book. The most recently published book of 39% of historians would not be included among their department's publishing totals using the 3-year timeframe. For most humanities disciplines, the median number of books per person published in a 5-year period is zero, reflecting their long gestation time. Short timeframes fail to capture the rhythm of book publication among humanist scholars. By comparison, the rhythm of conference proceeding publication in Computer Science is faster: 93% of computer scientists have at least one conference proceeding over 10-years, 89% over 5-years, and 86% over 3-years. Clearly, shorter timeframes have the potential to disadvantage some disciplines in a comparative context, particularly the humanities and other fields where books are a more common mode of knowledge dissemination. Considering only journal articles, shorter timeframes still disadvantage humanities disciplines, where articles are typically single-authored and relatively long (Fanelli and Glänzel, 2013). In German Language and Literature, for example, 88% of scholars authored a journal article over a 10-year period, 79% authored an article over a 5-year period, and 67% authored an article over a three-year period. The number of scholars whose work is captured decreases by 21% when the timeframe is reduced from 10- to 3-years. In Economics, the decline over the same timeframe is 7%, and in Physics the decline is 3%.

## Bibliometric Outputs and Faculty Career Stage

### Publishing Expectations Should Be Normalized on Career Stage

Academic departments and programs are often the units of analysis in bibliometric evaluations, and they are invariably composed of scholars at different career stages. Nonetheless, most comparative bibliometric data are not normalized on academic age. In the discipline History, for example, the mean year of terminal degree in departments at US research institutions spans approximately 20 years (Figure 1E); some departments are home to much younger faculty members than others. Professorial rank is also varied (Figure 1F); in some departments full professorships are the most common rank, while in others assistant professors represent more than 50% of faculty members. Publication productivity is known to differ across age cohorts as access to resources and many other factors change throughout the course of one's career (e.g., Kyvik, 1990; Davis et al., 2001; Abramo et al., 2011; Way et al., 2017; Savage and Olejniczak, 2021). Supplementary Table 4 separates publishing activity by rank, and substantial inter-rank variation is observed in most disciplines. For example, the median journal article count over 5-years for an assistant professor of Evolutionary Biology is 14 articles, while the median count for a full professor in the same field is 25 articles–a 78% increase (this holds true even when considering only those assistant professors who earned their terminal degree five or more years ago). In disciplines where book publishing is common, the median number of books authored by full professors is often two or three times the median number of books published by associate professors. The median number of books authored by assistant professors is almost always zero (mean values between 0.0 and 1.0), underscoring both the long gestation time of books and the importance of normalizing departmental publishing expectations against the career stages of the department's faculty members.

## What Becomes a Bibliometric Artifact?

Many important knowledge dissemination strategies are not registered as bibliometric artifacts with a persistent digital record (e.g., DOI, ISBN). These works are unlikely to be indexed in bibliometric databases. In some cases, a discipline does not create digital artifacts for some classes of work. Supplementary Table 1, for instance, indicates that humanists do not author many publications recorded as conference proceedings. Humanists at research universities regularly attend and submit their work to conferences, presenting that work in papers they read or circulate to other attendees. In the humanities, however, conference presentations much less frequently are published as conference proceedings, and therefore do not become bibliometric artifacts as often as they do in other fields. Other types of scholarship are also absent from most databases, including, e.g., choreographies, musical compositions, zines, blogs, datasets, and software. Some works are “small circulation ephemera” (e.g., zines as described by Brown et al., 2021), and archiving and cataloging these works may be antithetical to the ethos in which they were created. In other cases, ephemera are cataloged and preserved (e.g., Esling, 2013), but those archives are not widely used in academic evaluation. *For comparisons across disciplines to characterize the scholarship of faculty members in academic units, evaluators must move from asking “what journals does this database contain?” to questions such as “what knowledge dissemination types are pertinent to these disciplines?” and “do the types of knowledge production common in this discipline become quantifiable artifacts?”*

## Concluding Remarks

The number of journal articles (and the number of journals in which those articles are published) has increased markedly in the last few decades. In light of academe's growing evaluation culture—in which those publications are a de facto currency—we sought to describe ways to enhance evaluation of publishing activity across disciplines. Not all active scholars in any field will follow the same pattern of publication, and not all articles or books are of equal value: publication counts do not reflect quality. Citations to journal articles are sometimes considered a proxy for the impact or quality of an article, but citations to non-article publication types are difficult to procure, and we agree with the conclusions of many authors who reject the use of citations as quality indicators. Aksnes et al. (2019), for example, concluded that “…citation indicators seem of little help in the evaluation of the solidity/plausibility, originality, and societal value of research.” We echo the sentiments expressed in the Leiden Manifesto (Hicks et al., 2015); specifically we underscore that “Quantitative evaluation should support qualitative, expert assessment” and that academic unit evaluations should “Measure performance against the research missions of the institution, group or researcher.” Within the humanities, particularly among visual and performing arts, bibliometric measures may not be suitable for academic unit evaluation. Considering our comments earlier in this Opinion regarding “What becomes a bibliometric artifact?” we further caution against archiving and evaluating art scholarship as an alternative to bibliometrics (for broader discussions on archiving and quantifying visual and performing scholarship, see e.g., Piper, 1999; Jones, 2018). We recognize that bibliometrics are often used to create efficiencies in the evaluation process, but quantitative data should supplement qualitative assessment. Bibliometric data are not a replacement for human judgment, and scholarly publications represent only one group of tiles in the greater mosaic of a faculty member's activity. When bibliometric data *are* included in evaluations, they should represent accurately the differences among disciplines and career stages, be wider in scope than only journal articles, and incorporate discipline-specific rhythms of publication.

## Author Contributions

RW conceived the opinion article. AO created the data tables on which the opinion is based. All authors discussed the content and contributed to the final manuscript.

## Funding

Data and computational resources were provided by Academic Analytics, LLC.

## Conflict of Interest

AO and WS are paid employees of Academic Analytics, LLC, and RW is a paid consultant to Academic Analytics, LLC. None of the authors have an equity interest in Academic Analytics, LLC. The results presented reflect the authors' opinions, and do not necessarily reflect the opinions or positions of Academic Analytics, LLC. Academic Analytics, LLC management had no oversight or involvement in the project and were not involved in preparation or review of the manuscript. All work was done as part of the respective authors' research, with no additional or external funding.

## Publisher's Note

All claims expressed in this article are solely those of the authors and do not necessarily represent those of their affiliated organizations, or those of the publisher, the editors and the reviewers. Any product that may be evaluated in this article, or claim that may be made by its manufacturer, is not guaranteed or endorsed by the publisher.
