# Supplementary material for: The Rhythms of Scholarly Publication: Suggestions to Enhance Bibliometric Comparisons Across Disciplines
Source: Front Res Metr Anal. 2022 Jan 25;7:812312. doi: 10.3389/frma.2022.812312 (PMC8821823; doi:10.3389/frma.2022.812312)
Supplement: Supplementary file 5 [file Table_5.DOCX]

**Data source and method**

We extracted the discipline affiliations, professorial ranks, and publication histories of 198,989 scholars affiliated with 11,473 academic departments at 392 Ph.D. granting institutions in the United States from the Academic Analytics commercial database (database version AAD2019-1740, representing the 2019-2020 academic year). Academic Analytics produces annual database snapshots based on manual collection and disambiguation of faculty rosters (both department and Ph.D. program) from publicly available data sources, checking at least annually for faculty member name changes, new hires, retirements, rank promotions, and department, program, and university affiliation changes. This manual data collection process is supplemented by voluntary institutional validation of those rosters. Some institutional subscribers to Academic Analytics commercial dataset also submit faculty rosters which are then vetted against publicly available sources. Academic Analytics employs a semi-automated data matching process to associate each publication with its respective author(s), relying on third-party databases rather than self-reported data from, e.g., faculty-maintained CVs. Journal articles and conference proceedings metadata are acquired through CrossRef (<https://www.crossref.org/>); books and book chapters are from Baker and Taylor (<https://www.baker-taylor.com/>). We summed the number of authorships associated with each person in the dataset for each publishing type over three time periods: ten years (2010-2019), five years (2015-2019), and three years (2017-2019). Faculty members who had not earned their terminal degree before the beginning of a particular timeframe were not included in the summary counts for that timeframe. We also created a dummy variable indicating whether a faculty member had (1) or had not (0) authored a publication of each type in each of those four timeframes, facilitating calculation of the percent of faculty who participated in each publishing type during that timeframe. Departments in the Academic Analytics database are assigned to one or more of 170 disciplines based on National Center for Education Statistics (NCES) Classification of Instructional Programs (CIP) code classifications (NCES, 2013). A list of all universities and departments, as well as their mapping to the taxonomy of disciplines, is available among the supplemental materials.

All of the extracted data tables were ingested into RStudio (RStudio Team, 2020) and four discipline-level summary tables were produced using R version 4.1.0 (R Core Team, 2020). The summary tables are available as supplemental materials; the four tables describe the following characteristics of each discipline:

1. Supplemental Table 1: the total number of journal articles, conference proceedings, books, and book chapters produced by scholars in each discipline, and the percent of the overall disciplinary literature each of these publishing types represents (10-year total: 2010-2019);
2. Supplemental Table 2: for each discipline, the mean and median counts of publications per person in each publishing type over the most recent 3-, 5-, and 10-year timeframes (each ending in 2019);
3. Supplemental Table 3: the rate of participation in each publishing type in each discipline over the most recent 3-, 5-, and 10-year timeframes (ending in 2019); and
4. Supplemental Table 4: mean and median publications per person by discipline and professorial rank over the most recent 3-, 5-, and 10-year timeframes (ending in 2019).
